# In vitro activity of meropenem-vaborbactam combinations and eravacycline against carbapenem-resistant *Acinetobacter baumannii*

**DOI:** 10.1038/s41598-025-19642-y

**Published:** 2025-09-30

**Authors:** Amira H. El-Ashry, Ahmed Mostafa Abdrabou, Nora El-Tantawy, Rasha Mahmoud, Rasha El-Mahdy

**Affiliations:** 1 Department of Medical Microbiology and Immunology, Faculty of Medicine, Mansoura University, Mansoura, Egypt; 2 Department of Public Health, Faculty of Applied Medical Sciences, Al-Baha University, Al-Baha, Saudi Arabia; 3 Department of Medical Parasitology, Faculty of Medicine, Mansoura University, Mansoura, Egypt; 4 Department of Internal Medicine Department, Nephrology and Dialysis Unit, Mansoura University, Mansoura, Egypt

**Keywords:** CRAB, Eravacycline, Meropenem-vaborbactam, Antimicrobial combination, Antimicrobial resistance, Drug discovery, Microbiology

## Abstract

Treatment of carbapenem-resistant *Acinetobacter baumannii* (CRAB) presents a growing clinical challenge. This study evaluated the in vitro efficacy of eravacycline and the potential synergistic activity of meropenem-vaborbactam in combination with either gentamicin or ceftazidime against carbapenemase-producing *Acinetobacter baumannii* isolates. A total of 25 CRAB isolates were collected from different clinical samples. Antimicrobial susceptibility was determined via disc diffusion. Meropenem was tested by both disc diffusion and gradient strips. Polymerase chain reaction (PCR) was used to screen these isolates for the carbapenemase genes *bla*_*OXA-51,*_* bla*_*OXA-23,*_* bla*_*IMP*_*, bla*_*VIM*_*, bla*_*OXA-48*_*, bla*_*NDM*_ and *bla*_*KPC*_. Extensively drug-resistant (XDR) CRAB isolates were selected for evaluating colistin, eravacycline and the in vitro synergy of antimicrobial combinations via gradient strips for meropenem-vaborbactam, gentamicin, and ceftazidime. All CRAB isolates were sensitive to tigecycline and were either multidrug resistant or XDR. The minimum inhibitory concentrations (MIC50s and MIC90s) of meropenem were 32 μg/mL and 256 μg/mL, respectively. Among these genes, *bla*_*OXA-23*_ was the most prevalent gene. The MIC_50_ and MIC_90_ of colistin were 1 and 2 μg/mL, respectively. The MIC_50_ and MIC_90_ of eravacycline were 0.125 μg/mL and 0.5 μg/mL, respectively. Meropenem-vaborbactam in combination with ceftazidime or gentamicin showed synergy in 45.5% and 36.4% of the XDR isolates and additivity/indifference in 54.5% and 63.6% of them, respectively, with no antagonism. Our findings suggest that eravacycline, as well as combination therapies involving meropenem-vaborbactam with either gentamicin or ceftazidime, may offer promising therapeutic potential for CRAB infections pending further clinical evaluation. These agents demonstrated notable in vitro activity, including potential synergistic effects, particularly against isolates harboring carbapenemase enzymes.

## Introduction

Carbapenems are typically the best treatment for *Acinetobacter baumannii (A. baumannii*) infections. However, the undue use of these antibiotics has promoted the emergence of carbapenem-resistant strains^1^. Carbapenem-resistant *Acinetobacter baumannii* (CRAB), which commonly causes ventilator-associated pneumonia, bloodstream, wound, soft tissue, and urinary tract infections, can cause significant nosocomial infections worldwide with high mortality rates^1,2^. The World Health Organization (WHO) Priority Pathogens List for antibiotic research has identified CRAB as a priority 1-critical life-threatening nosocomial pathogen^3^. Resistance to carbapenem in *A. baumannii* has been attributed mainly to the production of carbapenemases. Other mechanisms include alteration of penicillin-binding proteins (PBPs), outer membrane protein loss, and excess production of the efflux pump^4^. The majority of CRAB strains are reported to be extensively drug resistant (XDR), meaning that they are resistant to at least one agent in all antibiotic categories. CRAB poses a significant therapeutic problem because of the limited therapeutic range of options. Existing treatments involve colistin, tigecycline, and high-dose sulbactam, which are frequently used in combination therapy, but there are significant constraints on these agents^5^. Colistin has been noted to have nephrotoxicity and poor lung penetration. Tigecycline has unpredictable efficacy, with a demonstration of lower efficacy in bloodstream infections. Sulbactam, although effective against *A. baumannii,* is usually affected by resistance to β-lactamases. These restrictions underscore the dire need for more effective and safer therapeutic alternatives. This background highlights the need to consider new drug combinations for treating CRAB^6^. Thus, new medication regimens are urgently needed to successfully treat infections caused by CRAB.

Eravacycline is a novel tetracycline molecule that resembles tigecycline in structure and, like other tetracyclines, binds to the 30S ribosomal subunit to prevent bacterial protein synthesis^7^. Ribosomal protection and acquired tetracycline efflux minimally affect eravacycline, positioning it as a viable treatment option for CRAB infection^8^.

Vaborbactam, a new serine-β-lactamase inhibitor, was recently introduced in combination with carbapenem (meropenem-vaborbactam) to prevent carbapenemase-mediated resistance by restoring susceptibility to β-lactams^9^. Meropenem-vaborbactam has been approved for the management of carbapenem-resistant *Enterobacterales* (CRE) infections^10^. However, there have been recent reports of resistance to meropenem-vaborbactam^11^. Thus, antimicrobial combinations have become effective therapeutic approaches that are frequently used to treat infections caused by resistant microorganisms^8,11^. In this context, evidence remains limited to the ideal antibiotic combinations to enhance the efficacy of meropenem–vaborbactam and circumvent the emergence of antimicrobial resistance. Although vaborbactam does not target the class B and D carbapenemases common in *A. baumannii*, its inclusion in combination regimens underscores the potential of repurposing β-lactamase inhibitors beyond their typical spectrum through synergistic effects, warranting continued experimental evaluation.The purpose of this study was to evaluate the in vitro efficacy of eravacycline and assess the potential synergistic effects of meropenem-vaborbactam when combined with either ceftazidime or gentamicin against a panel of carbapenem-resistant *Acinetobacter baumannii* (CRAB) isolates.

## Methods

A descriptive hospital-based cross-sectional study was conducted in the Medical Microbiology and Infection Control Unit (MDICU) at Mansoura University Hospital in Egypt. The Mansoura Faculty of Medicine’s Institutional Review Board (IRB), code number R.25.05.3186, approved the collection of bacterial isolates and the use of patient medical records with informed consent. All methods were performed according to the relevant guidelines and regulations. This research included a total of 25 nonduplicate CRAB isolates previously collected from May 2024 to April 2025. The isolates were collected from blood, sputum, and wound exudate samples from patients admitted to different wards of Mansoura University Hospitals (MUHs), and they were originally identified by phenotypic methods concerning standard microbiological laboratory procedures as gram-negative coccobacilli with nonhemolytic growth on blood agar; pink to light purple growth on MacConkey agar medium at 37 °C; positive catalase tests; negative oxidase tests; nonfermentative (oxidative) tests for glucose; negative indoles; positive methyl red; negative Voges-Proskauer; positive citrate; negative urease tests; nonfermentative (alkaline/alkaline) triple sugar iron; and growth on nutrient agar at 44 °C^12^.

### Testing for antimicrobial susceptibility

All *A. baumannii* isolates were tested via antimicrobial susceptibility testing (AST) on Mueller‒Hinton agar via the Kirby–Bauer method against piperacillin (100 μg), ceftazidime (30 μg), cefepime (30 μg), aztreonam (30 μg), meropenem (10 μg), imipenem (10 μg), tobramycin (10 μg), gentamicin (10 μg), levofloxacin (10 μg), ciprofloxacin (5 μg), and tigecycline (15 μg) (Liofilchem, Roseto Degli Abruzzi, Italy) in accordance with Clinical and Laboratory Standards Institute (CLSI) guidelines^13^. Tigecycline breakpoints from the FDA were used because the CLSI does not provide interpretive criteria for tigecycline against *Enterobacterales.* Therefore, FDA breakpoints were adopted as the available and appropriate reference^14^.

The E test, which specifies a minimum inhibitory concentration (MIC) of ≤ 2 μg/mL for susceptibility and ≥ 8 μg/mL for resistance, was used. Isolates exhibiting resistance to any tested carbapenem were potentially identified as carbapenem resistant and subsequently selected for meropenem MIC determination^13^. Standard criteria established for *Acinetobacter* species^15^ were employed to identify the multidrug-resistant (MDR) and extensively drug-resistant (XDR) status of the isolates. The XDR CRAB isolates were selected for evaluating eravacycline and the in vitro synergy of antimicrobial combinations with E-test strips for meropenem-vaborbactam (MEV 0.016/8–256/8 µg/mL), gentamicin and ceftazidime (MTS™ Liofilchem).

Because CLSI breakpoints are not available, the European Committee on Antimicrobial Susceptibility Testing (EUCAST) MIC breakpoints published for *Enterobacterales* were employed to determine susceptibilities to eravacycline: susceptible ≤ 0.5 μg/mL and resistant > 0.5 μg/mL for comparison^16^.

Colistin susceptibility testing was performed via the broth microdilution method with colistin sulfate powder (Acros Organics BVBA, Geel, Belgium). EUCAST MIC breakpoints were employed to determine susceptibility MICs ≤ 2 μg/mL for susceptible strains and > 2 μg/mL for resistant strains^16^.

### Phenotypic and genotypic detection of CRAB

Phenotypic methods, such as the modified carbapenem inactivation method (mCIM)/EDTA-modified carbapenem inactivation method (eCIM), were employed to assess the capacity of each carbapenem-resistant isolate to generate carbapenemases and metallo-β-lactamases (MBLs)^13^. To determine the presence of the carbapenemase genes *bla*_*OXA-51,*_* bla*_*OXA-23,*_* bla*_*IMP*_*, bla*_*VIM*_*, bla*_*OXA-48*_*, bla*_*NDM*,_ and *bla*_*KPC*_, the results of the phenotypic methods were validated via multiple rounds of PCR as previously reported^17,18^. The DNA of CRAB isolates with phenotypic confirmation was extracted via the boiling method^19^ and then used as a template for PCR with appropriate primers for the *bla*_*OXA-51*_, *bla*_*OXA-23,*_* bla*_*IMP*_*, bla*_*VIM*_*, bla*_*OXA-48*_*, bla*_*NDM*,_ and *bla*_*KPC*_ genes. The primers used in the study are listed in Table 1.

**Table 1:** Sequences of Primers used for carbapenemase gene detection.

Gene	Sequence (5′–3′)	Product size (bp)	References
*bla*_*OXA−51*_	TAATGCTTTGATCGGCCT TG TGGATTGCACTTCATCTTGG	353	^17^
*bla*_*OXA−23*_	GATCGGATTGGAGAACCAGA ATTTCTGACCGCATTTCCAT	501	^17^
*bla*_*IMP*_	GGAATAGAGTGGCTTAAYTCTC GGTTTAAYAAAACAACCACC	232	^18^
*bla*_*VIM*_	GATGGTGTTTGGTCGCATA CGAATGCGCAGCACCAG	390	^18^
*bla*_*OXA−48*_	GCGTGGTTAAGGATGAACAC CATCAAGTTCAACCCAACCG	438	^18^
*bla*_*NDM*_	GGTTTGGCGATCTGGTTTTC CGGAATGGCTCATCACGATC	621	^18^
*bla*_*KPC*_	CGTCTAGTTCTGCTGTCTTG CTTGTCATCCTTGTTAGGCG	798	^18^

### Perpendicular E test for testing the synergistic effects of antimicrobial combinations

Several two-drug combinations were tested for their synergistic effects on XDR CRAB isolates via perpendicular E tests in triplicate. E-test strips containing meropenem-vaborbactam and either gentamicin or ceftazidime were positioned perpendicular to each other over a lawn of the representative bacterial isolate on Mueller Hinton agar plates for 18 h at 37 °C^20^.

The fractional inhibitory concentration index (FICI) was calculated via the following formula: FICA + FICB = FICI, where FICA represents the MIC of drug A in combination divided by the MIC of drug A alone, and FICB represents the MIC of drug B in combination divided by the MIC of drug B alone. The FICIs were interpreted as follows: antagonistic effect (≥ 4), additive/indifferent effect (0.5–4), and synergistic effect (≤ 0.5)^21^.

**Fig. 1:**
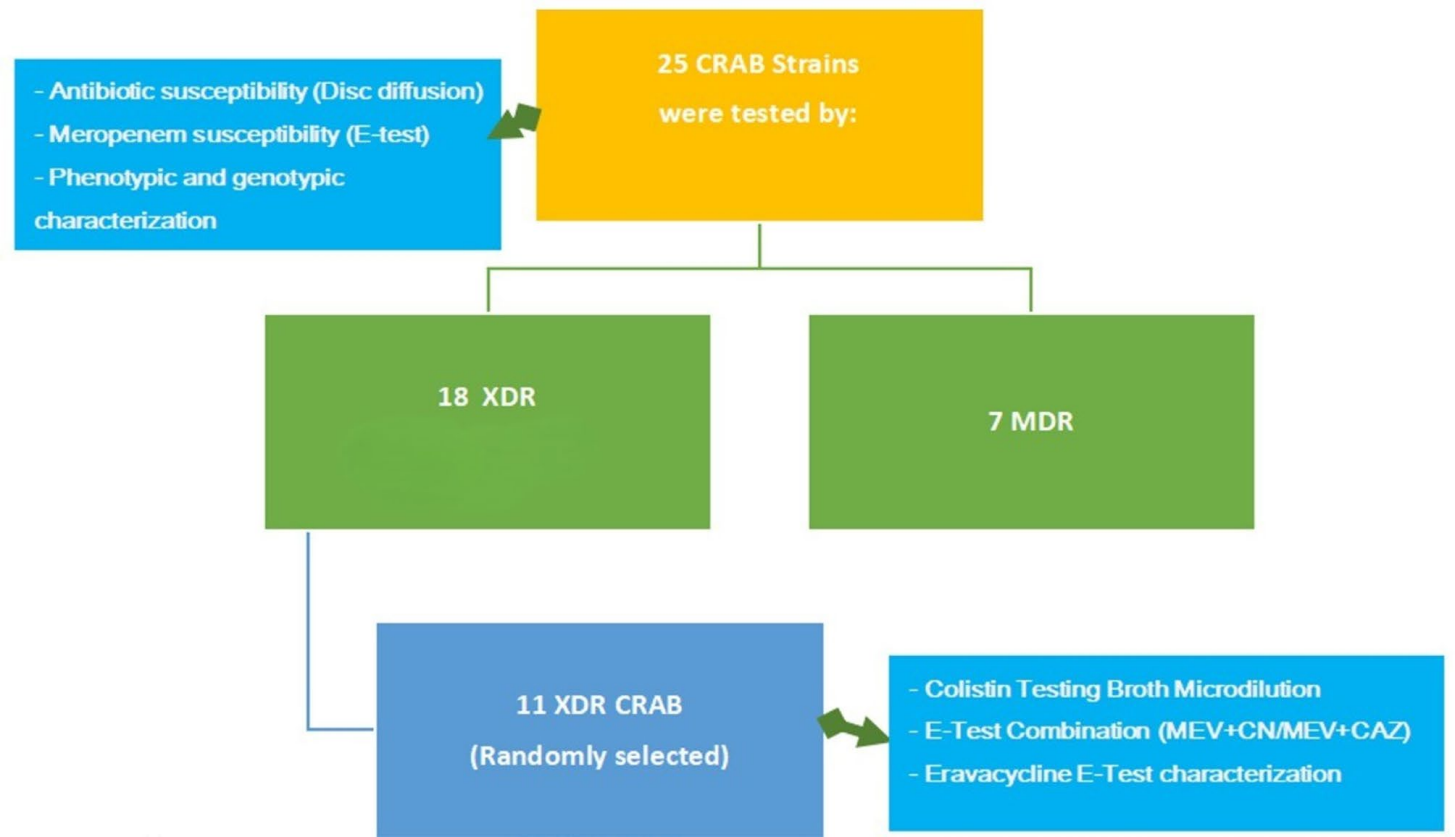
Flowchart depicting the experimental design.

For quality assurance, an *Escherichia coli* (*E. coli*) strain (ATCC 25922) and *Klebsiella pneumoniae* ATCC BAA-2146 were used. The flowchart depicting the experimental design is shown in Fig. 1.

### Data statistical analysis

Statistical Package for the Social Sciences (SPSS) software (version 25.0)^22^ was used for descriptive analysis to calculate the mean, median, MIC_50_, and MIC_90_ for the tested antibiotics.

## Results

A total of 25 nonduplicate CRAB isolates were collected from patients with hospital-acquired pneumonia (56%), wound infection (28%), and bloodstream infection (16%) in Mansoura University Hospitals, Mansoura, Egypt. The median age of patients with CRAB infections was 35 years (range: 1–69 years), and thirteen were female. Both Disk diffusion and E-tests revealed that all twenty-five strains were resistant to meropenem, with MIC_50_ and MIC_90_ values of 32 μg/mL and 256 μg/mL, respectively.

**Fig. 2:**
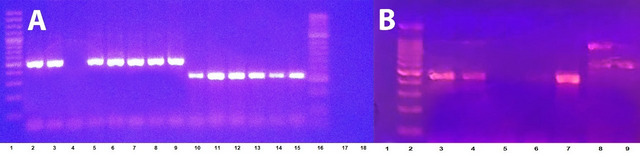
PCR results for carbapenemase genes. (**A**) Agarose gel electrophoresis of *blaOXA-*23 and *blaOXA-*51 amplicons. Lanes (1, 16): DNA ladder 100 bp; lanes (2, 3, 5–9) for the *blaOXA-23* gene (501 bp); lanes (10–15) for the *blaOXA-51* gene (353 bp); negative control in lanes (17, 18) and (**B**) agarose gel electrophoresis of *bla*OA-48, *blaNDM, and blaKPC*; lane 1: negative control; lane 2: DNA ladder 100 bp; lanes (3, 4, 7): *blaOXA-48* (438 bp); lane 8: *blaNDM, blaKPC,* (621 bp, 798 bp); positive control in lane 9 (*Klebsiella pneumoniae* ATCC BAA-2146); and NDM 621 bp.

All the isolates (100%) had the *bla*_*OXA-*51_ gene, confirming that they were *A. baumannii*. Other carbapenemase genes were distributed in 24 CRAB isolates as follows: 14 isolates (56%) assessed positive for *bla*_*OXA-23*_; *bla*_*NDM*_ was acquired in seven isolates (28%); *bla*_*OXA-48*_ was present in four isolates (16%); and only two isolates (8%) were positive for *bla*_KPC_. No isolates carried *bla*_*IMP*_ or *bla*_*VIM*_ genes. Two (8%) CRAB isolates harbored more than one carbapenemase gene, and the combinations observed were *bla*_*OXA-23*_ + *bla*_*NDM*_ and *bla*_*NDM*_ + *bla*_*KPC,*_ each in one isolate. (Fig. 2A, B).

The identification of carbapenemase enzymes via phenotypic methods and PCR tests showed a good degree of concordance. When mCIM combined with eCIM was compared with gene detection, the coincidence rate in the present study was 95.8% (23/24), except for one false-negative mCIM, in which the *bla*_OXA*-23*_ gene was detected via PCR. The sensitivity and specificity of the mCIM combined with the eCIM were 95.8% and 100%, respectively.

All CRAB isolates were MDR (100%). Eighteen (72%) CRAB isolates were XDR and resistant to all the tested antibiotics except tigecycline, as determined by the disk diffusion method. Among the 18 XDR CRAB isolates, eleven were randomly chosen and stratified by the source of isolation to ensure proportional representation of different clinical specimen types (sputum, blood, and wound). Eravacycline was evaluated against 11 randomly chosen XDR CRAB isolates, which presented MIC_50/90_ values of 0.125 μg/mL and 0.5 μg/mL, respectively. The MIC_50/90_ values of colistin, which was used as a comparator antimicrobial, were 1/2. Table 2 summarizes the MIC of colistin and the findings of the synergy assay, which was performed to determine the MIC values and in vitro synergism of the individual antibiotic combinations against 11 typical XDR CRAB isolates.

**Table 2:** Minimum inhibitory concentrations of antibiotics and antibiotic combinations FICI for 11 XDR CRAB isolates.

Sample number	Sample origin	Sex	Age	MICs of individual antimicrobials (µg/mL)					FICI combination (MEV + CN)	FICI combination (MEV + CAZ)
				COL	MRP	MEV	CN	CAZ		
1	Wound	F	22	2	96	96	256	256	1.66	2
2	Sputum	F	27	0.5	48	48	256	192	1.25	0.5
3	Blood	M	1	2	256	256	128	256	0.44	0.43
4	Wound	M	38	0.5	16	16	256	256	0.5	0.28
5	Blood	M	1	1	24	24	256	256	1.04	0.171
6	Sputum	F	20	0.125	16	16	32	128	0.5	0.44
7	Blood	F	1	0.125	32	16	64	256	0.31	0.28
8	Sputum	F	41	2	64	96	128	256	1.25	1.17
9	Sputum	F	56	1	16	16	96	256	2	0.875
10	Wound	F	40	1	32	32	64	96	2	2
11	Sputum	F	50	4	256	256	256	256	2	2

Eleven XDR CRAB isolates were tested for the meropenem-vaborbactam-gentamicin combination, which exhibited 63.6% (7/11) additivity/indifference (FICI range = 1.04–2) and 36.4% (4/11) synergy (FICI range = 0.31–0.5); no antagonism was detected. All the isolates had a mean FICI of 1.18 (range 0.31–2), suggesting antimicrobial interactions, as depicted in Table 3.

**Table 3:** Synergy results for different antimicrobial combinations.

	XDR CRAB (*N* = 11)		
Antibiotic	Synergistic %	Additive/Indifferent %	Antagonistic %
MEV + CN	36.4% (4/11)	63.6% (7/11)	0
MEV + CAZ	54.5% (6/11)	45.5% (5/11)	0

MEV: meropenem-vaborbactam, CN: gentamicin, CAZ: ceftazidime.

Verification of the combination of meropenem and vaborbactam with ceftazidime against the same 11 XDR CRAB isolates yielded 54.5% (6/11) synergy (FICI range = 0.17–0.5) and 45.5% (5/11) additivity/indifference (FICI range = 0.88–2). The total FICI mean was 0.9 (range 0.17–2), supporting the combination interaction. No isolates had an FICI ≥ 4.

The geometric median MIC for meropenem-vaborbactam decreased from 32 to 24 μg/mL, and that for gentamicin decreased from 128 to 96 μg/mL when tested in combination via perpendicular E-test assays. The median MICs for meropenem-vaborbactam and ceftazidime decreased from 32 to 12 μg/mL and from 256 to 64 μg/mL, respectively, when meropenem-vaborbactam was combined with ceftazidime, as shown in Table 3.

Pair-isolate combinations with sensitive MIC breakpoints were achieved in 3 of 11 (27.3%) isolates for meropenem-vaborbactam (≤ 8 μg/mL) combined with gentamicin (≤ 4 μg/mL) and in 4 (36.4%) isolates for meropenem-vaborbactam with ceftazidime (≤ 8 μg/mL), as shown in Table 4.

**Table 4:** Minimum inhibitory concentration (MIC) ranges, MIC50s, and MIC90s of 11 XDR CRAB isolates for the tested antimicrobial agents.

				XDR CRAB (*N* = 11)		
Antibiotic	S	I	R	Range µg/mL	MIC50	MIC90
COL	10		1	0.125-4	1	2
MRP			11	16–256	32	256
ERV	11			0.047-0.5	0.125	0.5
MEV			11	16–256	32	256
CN			11	32–256	128	256
CAZ			11	96–256	256	256
MEV in MEV + CN combination	3		8	2-256	24	256
CN in MEV + CN combination	0	2	9	8-256	96	256
MEV in MEV + CAZ combination	4		7	1-256	12	224
CAZ in MEV + CAZ combination	2	1	8	6-256	64	256

MIC: Minimal inhibitory concentration, COL: Colistin, MRP: Meropenem, ERV: Eravacycline, CN: gentamicin, MEV: meropenem -vaborbactam, CAZ: ceftazidime, S: sensitive; I: intermediate; R: resistant.

**Fig. 3:**
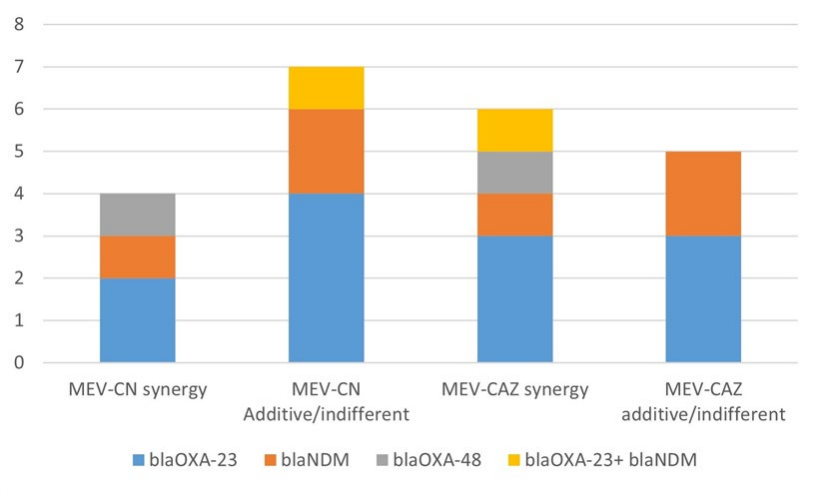
In vitro synergistic effects of different antimicrobial combinations on 11 XDR CRAB isolates, where MEV-CN indicates meropenem-vaborbactam-generate and where MEV-CAZ indicates meropenem-vaborbactam-ceftazidime.

In particular, the KPC, MBL, and MBL + KPC strains presented synergy rates of 42.9% (3/7), 33.3% (1/3), and 0% (0/1), respectively, with the meropenem-vaborbactam/gentamicin combination. A somewhat higher rate of synergy was detected for the KPC strains (57.1%, 4/7), and a similar rate of synergy was detected for MBL (33.3%, 1/3) in the meropenem-vaborbactam/ceftazidime combination, whereas the strains with both carbapenemase types exhibited 100% synergy (1/1), as demonstrated in Fig. 3.

## Discussion

The notable persistence and antibiotic resistance of *A. baumannii* make it one of the ESKAPE bacteria prioritized by the WHO for novel treatment development^23^. It has recently drawn increased attention because of its high propensity to develop antibiotic resistance, notably to carbapenems, and the emergence of CRAB isolates has become a major global problem^24^. This study aimed to assess the in vitro activity of eravacycline and evaluate the potential synergistic effects of meropenem-vaborbactam when combined with either ceftazidime or gentamicin against carbapenem-resistant *Acinetobacter baumannii* (CRAB) isolates. By identifying effective combinations, this study sought to explore alternative therapeutic strategies that could enhance antimicrobial efficacy and potentially curb resistance development in this highly drug-resistant pathogen.

In the present study, *A. baumannii* was isolated from various clinical samples, including sputum, blood, and surgical wounds, which is consistent with findings from two previous studies in Jordan and Nigeria, reported *A. baumannii* isolation from numerous clinical samples^25,26^. This may be attributed to its ability to persist for extended periods in hospital settings, adapt to specific environmental pressures, and its non-fastidious nature^27^. The majority of the CRAB isolates in this study (56%) were from sputum samples, given that *A. baumannii* is a respiratory colonizer, followed by surgical wounds (28%). Research conducted in the same region of Egypt supported this result^28^. Similarly, other studies^29,30^ reported respiratory samples as the primary source of *A. baumannii* isolates.

All *A. baumannii* isolates, whether carbapenem resistant or carbapenem sensitive, typically harbor the *bla*_*OXA-*51_ gene. Carbapenem resistance, particularly imipenem resistance, develops only in isolates with overexpression of the insertion sequence (ISAbaI) upstream of *bla*_*OXA-*51_. Consequently, it serves as a species identification marker rather than a resistance marker^31^. All the CRAB isolates studied harbored *bla*_*OXA-*51_, which was per previous studies in Egypt^32,33^. As previously mentioned, class D (oxacillinase) and class B (MBL) carbapenemases are the primary contributors to carbapenem resistance in *A. baumannii* strains. This pathogen has also recently been reported to harbor class A *Klebsiella pneumoniae* carbapenemase (KPC)^1^, a finding supported by our results.

The *bla*_*OXA-23*_ gene was the most commonly detected carbapenemase in our CRAB isolates (56%). This finding agrees with previous studies in Egypt^34,35^ and elsewhere^36,37^, which reported that 50%, 70.5%, 92% and 94% of the *A. baumannii* isolates carried the *bla*_*OXA-*23_ gene, respectively. This result is also consistent with the global *bla*_*OXA-*23_ epidemiology, which can be explained by the ease with which the *bla*_*OXA-*23_ carbapenemase gene spreads between strains via lateral gene transfer^29^. The close distribution of the metallo-β-lactamase *bla*_*NDM*_ gene was previously reported^31^. Interestingly, a prior study reported that the drug resistance-associated genes *bla*_*IMP*_ and *bla*_*VIM*_ were absent, which was in line with our results^38^.

In our study, when the phenotypic and genomic levels for carbapenemase enzyme identification were compared, mCIM combined with eCIM demonstrated a sensitivity and specificity of 95.8% and 100%, respectively. Similarly, the combined mCIM and eCIM test provided crucial clinical signals for detecting carbapenemase-producing *Enterobacterales* in our previous report^39^ and another study, with approximately 92% sensitivity^40^.

There was an increased incidence of the XDR trait in the CRAB isolates (72%), which were resistant to all tested antibiotics apart from tigecycline. This aligns with previous Egyptian reports, whereas all *Acinetobacter* isolates were resistant to all antibiotics, except colistin and tigecycline, against which no resistance was recorded^41^. Recently, third-generation tetracycline eravacycline was introduced to address common bacterial tetracycline resistance mechanisms. All 11 XDR strains tested in our study had MIC_50/90_ values of 0.125 μg/mL and 0.5 μg/mL, respectively, for eravacycline, indicating its considerable effectiveness against our isolate collection. These ranges are consistent with those reported in earlier studies ^42,43^. According to Deolankar et al.^44^, all isolates, including XDR strains, had average MICs < 4 μg/mL for eravacycline, suggesting that eravacycline could be a potent therapeutic choice for infections caused by MDR or XDR *A. baumannii.* The low MIC_90_ of eravacycline against CRAB was reported previously^45^. Therefore, integrating eravacycline into the standard screening procedure used during specimen workup in clinical microbiology labs may be beneficial^44^.

Although eravacycline is therapeutically efficient against infections caused by gram-negative organisms and is well tolerated by patients, it should be safe for extremely resistant bacterial infections and utilized infrequently in clinical practice^46^. Meropenem-vaborbactam antimicrobial therapy offers promising alternatives to current agents for treating severe CRE infections. However, certain mutations have recently been shown to confer resistance to meropenem-vaborbactam among *Enterobacterales* in a few reports^10^.

The MIC range of meropenem-vaborbactam against our CRAB collection ranged from 16 to 256 μg/mL. This can be explained by the primary activity of meropenem-vaborbactam against microorganisms producing Class A and C carbapenemases, which were less prevalent in our study than its limited activity against Class D and B carbapenemase-producing microorganisms^46^, which were predominant in the present study. Harmonized, meropenem-vaborbactam and ceftazidime-avibactam have demonstrated limited efficacy against CRE isolates in a recent study in Egypt, with susceptibility rates of 14.3% and 31.4%, respectively^47^.

Higher sensitivity rates (48%, 58%) to meropenem-vaborbactam than those reported in our study were detected in the few Egyptian studies assessing susceptibility to it^39,48^. Hence, meropenem-vaborbactam antibiotics do not target metallo-β-lactamases or oxacillinases, the most common carbapenemases; combining existing antimicrobial drugs has become a key strategy in treating infections caused by carbapenemase-producing pathogens^49^.

The use of combination therapy may be beneficial in treating multidrug-resistant infections. However, combinations may have indifferent, antagonistic, or synergistic effects on clinical isolates. Moreover, the use of potentially toxic agents such as colistin in combination may challenge safe drug administration. Therefore, the use of β-lactam/β-lactamase inhibitor agents such as meropenem/vaborbactam is considered preferable^50,51^.

The results of the perpendicular E test in this study demonstrated that meropenem-vaborbactam in combination with gentamicin had a 63.7% additive/indifferent effect and a 36.4% synergistic effect with no antagonism against CRAB isolates. The combination of meropenem-vaborbactam with gentamicin against KPC-producing *Klebsiella pneumoniae* (*K. pneumoniae*) strains also showed additive (88.9%) and synergistic (11.1%) effects, according to the only study that tested this combination to our knowledge^11^. This finding was in line with our results, where the meropenem-vaborbactam/gentamicin combination was more additive than synergistic.

Similarly, the checkerboard assay demonstrated the synergistic effect of meropenem and several aminoglycosides against CR *E. coli* according to Terbtothakun et al., where meropenem plus gentamicin had the greatest synergistic effect (84.2%) among all aminoglycosides tested^49^.

Similarly, a recent case report of post-neurosurgical meningitis caused by carbapenemase-producing *K. pneumoniae* showed that antibacterial systemic combined therapy of meropenem-vaborbactam and gentamicin exhibited synergistic effects^52^. This result could be explained by the potential of meropenem and aminoglycoside combinations to induce membrane rupture, as aminoglycosides alter the outer membrane structure of gram-negative bacteria. Therefore, when aminoglycosides are combined with other antibiotics, such as carbapenems, their permeabilizing effect and periplasmic target site penetration increase, resulting in the restoration of meropenem’s in vitro activity^53^.

In our study, the combination of meropenem-vaborbactam and ceftazidime had synergistic effects on 6 out of 11 (54.5%) XDR CRAB isolates, while the remaining isolates exhibited additive or indifferent activity (45.5%). No antagonistic interactions were observed. Similar results were obtained when the same combination was tested against KPC-producing *K. pneumoniae* strains, with in vitro synergies of 33.3% and 66.7% additive interactions^11^. This may be attributed to the increased affinity of bacterial penicillin-binding proteins (PBPs) for meropenem and ceftazidime, resulting in the restoration of carbapenem activity^54^.

*Kuai *et al. reported synergistic interactions of ceftazidime-avibactam plus meropenem in checkerboard assays for 93.8% (15/16) of CRE strains^52^. In Egypt, the FICI method revealed synergistic and additive effects of the combination of ceftazidime-avibactam with meropenem against 2.5% and 55% of carbapenemase-producing *K. pneumoniae* isolates, respectively^55^.

A previous study revealed that the combination of meropenem and aminoglycosides has synergistic effects in combatting carbapenem-resistant *K. pneumoniae* strains that produce KPC-2 and NDM-1. This agreed with the meropenem-vaborbactam/gentamicin combination in our study, which had synergistic effects on 42.9% and 33.3% of the KPC and MBL strains, respectively, as gentamicin is not hydrolyzed by carbapenemases, thus restoring susceptibility to meropenem^56^.

This study has several limitations to acknowledge: (1) the small sample size (25 CRAB isolates) impacts generalizability; (2) the single-center design may not reflect regional variability; (3) molecular typing to assess clonal relationships was not performed; and (4) the use of EUCAST breakpoints for *Enterobacterales* to interpret eravacycline MICs in *Acinetobacter baumannii*, owing to the lack of CRAB-specific breakpoints. This extrapolation may lead to interpretive bias, as pharmacodynamic parameters and MIC distributions differ between *Enterobacterales* and non-fermenters. Readers should interpret eravacycline susceptibility results with this consideration in mind. (5) Animal or clinical studies are recommended to validate the results, as in vitro procedures might not reflect the clinical outcome.

In summary, our findings suggest that combining either eravacycline or meropenem-vaborbactam with ceftazidime or gentamicin offers promising therapeutic potential for CRAB infections, but further clinical evaluation is needed. Nevertheless, further research is necessary to validate their clinical efficacy and mechanism of action.

## Data Availability

All the data generated or analyzed during this study are included in this published article.
